# Effects of chemotherapy on contralateral breast cancer risk in *BRCA1* and *BRCA2* mutation carriers: A nationwide cohort study

**DOI:** 10.1016/j.breast.2021.12.007

**Published:** 2021-12-14

**Authors:** Delal Akdeniz, Mark van Barele, Bernadette A.M. Heemskerk-Gerritsen, Ewout W. Steyerberg, Michael Hauptmann, HEBON Investigators, Irma van de Beek, Klaartje van Engelen, Marijke R. Wevers, Encarnacion B. Gómez García, Margreet G.E.M. Ausems, Lieke P.V. Berger, Christi J. van Asperen, Muriel A. Adank, Margriet J. Collée, Denise J. Stommel-Jenner, Agnes Jager, Marjanka K. Schmidt, Maartje J. Hooning

**Affiliations:** a Department of Medical Oncology, Erasmus MC Cancer Institute, Rotterdam, the Netherlands; b Department of Public Health, Erasmus MC, Rotterdam, the Netherlands; c Department of Biomedical Data Sciences, Leiden University Medical Centre, Leiden, the Netherlands; d Institute of Biostatistics and Registry Research, Brandenburg Medical School Theodor Fontane, Neuroppin, Germany; e Department of Clinical Genetics, Amsterdam UMC, University of Amsterdam, Amsterdam, the Netherlands; f Department of Clinical Genetics, Amsterdam UMC, Vrije Universiteit Amsterdam, Amsterdam, the Netherlands; g Department for Clinical Genetics, Radboud University Medical Centre, Nijmegen, Netherlands; h Department of Genetics, Maastricht University Medical Centre, Maastricht, Netherlands; i Division of Laboratories, Pharmacy and Biomedical Genetics, Department of Genetics, University Medical Centre Utrecht, Utrecht, Netherlands; j Department of Genetics, University of Groningen, University Medical Centre Groningen, Groningen, Netherlands; k Department of Clinical Genetics, Leiden University Medical Centre, Leiden, the Netherlands; l Family Cancer Clinic, The Netherlands Cancer Institute, Antoni van Leeuwenhoek Hospital, Amsterdam, the Netherlands; m Department of Clinical Genetics, Erasmus University Medical Centre, Rotterdam, the Netherlands; n Division of Psychosocial Research and Epidemiology, Netherlands Cancer Institute, Amsterdam, the Netherlands; o Division of Molecular Pathology, Netherlands Cancer Institute, Amsterdam, the Netherlands

**Keywords:** PBC, primary breast cancer, CBC, contralateral breast cancer, HEBON, Hereditary Breast and Ovarian cancer research Netherlands, NCR, Netherlands Cancer Registry, PALGA, The nationwide network and registry of histo- and cytopathology in The Netherlands, HR, hazard ratio, CI, confidence interval, CMF, cyclophosphamide, methotrexate, 5-fluorouracil, Breast cancer, Secondary, Risk factors, Chemotherapy, BRCA1, BRCA2

## Abstract

**Aim:**

*BRCA1/2* mutation carriers with primary breast cancer (PBC) are at high risk of contralateral breast cancer (CBC). In a nationwide cohort, we investigated the effects of chemotherapeutic agents given for PBC on CBC risk separately in *BRCA1* and *BRCA2* mutation carriers.

**Patients and methods:**

*BRCA1* or *BRCA2* mutation carriers with an invasive PBC diagnosis from 1990 to 2017 were selected from a Dutch cohort. We estimated cumulative CBC incidence using competing risks analysis. Hazard ratios (HR) for the effect of neo-adjuvant or adjuvant chemotherapy and different chemotherapeutic agents on CBC risk were estimated using Cox regression.

**Results:**

We included 1090 *BRCA1* and 568 *BRCA2* mutation carriers; median follow-up was 8.9 and 8.4 years, respectively. Ten-year cumulative CBC incidence for treatment with and without chemotherapy was 6.7% [95%CI: 5.1–8.6] and 16.7% [95%CI: 10.8–23.7] in *BRCA1* and 4.8% [95%CI: 2.7–7.8] and 16.0% [95%CI: 9.3–24.4] in *BRCA2* mutation carriers, respectively. Chemotherapy was associated with reduced CBC risk in *BRCA1* (multivariable HR: 0.46, 95%CI: 0.29–0.74); a similar trend was observed in *BRCA2* mutation carriers (HR: 0.63, 95%CI: 0.29–1.39). In *BRCA1*, risk reduction was most pronounced in the first 5 years (HR: 0.32, 95%CI: 0.17–0.61). Anthracyclines and the combination of anthracyclines with taxanes were associated with substantial CBC risk reduction in *BRCA1* carriers (HR: 0.34, 95%CI: 0.17–0.68 and HR: 0.22, 95%CI: 0.08–0.62, respectively).

**Conclusion:**

Risk-reducing effects of chemotherapy are substantial for at least 5 years and may be used in personalised CBC risk prediction in any case for *BRCA1* mutation carriers.

## Highlights

• Contralateral breast cancer (CBC) risk is high in *BRCA1/2* mutation carriers.
• Chemotherapy for primary breast cancer results in decreased CBC risk in *BRCA1.*
• Anthracyclines with/without taxanes show the largest CBC risk reduction in *BRCA1.*
• For *BRCA2* similar trends are observed as in *BRCA1* mutation carriers.
• Chemotherapy must be considered in personalised CBC risk models.

## 1 Introduction

Women with a primary breast cancer (PBC) diagnosis and a pathogenic germline mutation in the *BRCA1* or *BRCA2* gene are at increased risk of developing metachronous contralateral breast cancer (CBC). The annual risk of CBC is around 1–3%, with young *BRCA1* mutation carriers having the highest risk [1,2]. *BRCA1/2* mutation carriers with PBC may opt for a contralateral risk-reducing mastectomy to reduce the risk of CBC, potentially improving survival [3].

In sporadic PBC patients a reduction in CBC risk is found after treatment with adjuvant endocrine treatment and/or adjuvant chemotherapy for PBC [4,5]. In *BRCA*-associated breast cancer the ability to repair double-strand DNA breaks is impaired because of insufficient homologous recombination repair function of the BRCA protein [[6], [7], [8], [9]]. Therefore, chemotherapeutics that cause double-strand DNA breaks (i.e. platinum salts, anthracyclines) are considered to be more effective. By eliminating precancerous cells or preclinical cancers, double-strand DNA breaks-inducing chemotherapeutics may reduce the occurrence of CBC in *BRCA1/2* mutation carriers.

So far, the effects of chemotherapy on CBC risk in *BRCA1/2* mutation carriers have been investigated only in a limited number of studies [1,10,11]; in only one study the effects of different chemotherapeutic agents on CBC risk were investigated, though with *BRCA1* and *BRCA2* mutation carriers combined [11]. *BRCA1*-associated tumours are however biologically different from *BRCA2*-associated breast tumours, and should therefore be studied separately [1,12,13]. Investigating the effects of different chemotherapy agents could prove useful for personalised CBC risk prediction and management.

In a large Dutch cohort, we therefore aimed to investigate the effects of chemotherapy overall and for various agents on CBC risk, separately for *BRCA1* and *BRCA2* mutation carriers.

## 2 Patients and Methods

Eligible patients were selected from the Hereditary Breast and Ovarian cancer research Netherlands (HEBON) cohort [14]. The HEBON study is an ongoing Dutch nationwide collaboration that aims to include all members from breast and/or ovarian cancer families tested for a *BRCA1/2* mutation, recently extended for pathogenic mutations in *CHEK2, PALB2* and *ATM*. These women have been identified through all eight Clinical Genetics centres in the Dutch University Medical Centres and the Netherlands Cancer Institute. Approval from the Medical Ethics Committees of all participating centres was obtained. Written informed consent was provided by all participating women, or either a close relative or proxy in case of a deceased individual. From January 1999 onwards, data on patient, tumour, (preventive) treatment, and follow-up characteristics are collected and updated by linkage to the Netherlands Cancer Registry (NCR) and the The nationwide network and registry of histo- and cytopathology in The Netherlands (PALGA). In addition, regular linkage with the Municipal Administrative Database provides updated information on vital status. The latest follow-up date in this study is December 31, 2017.

**Fig. A.1:**
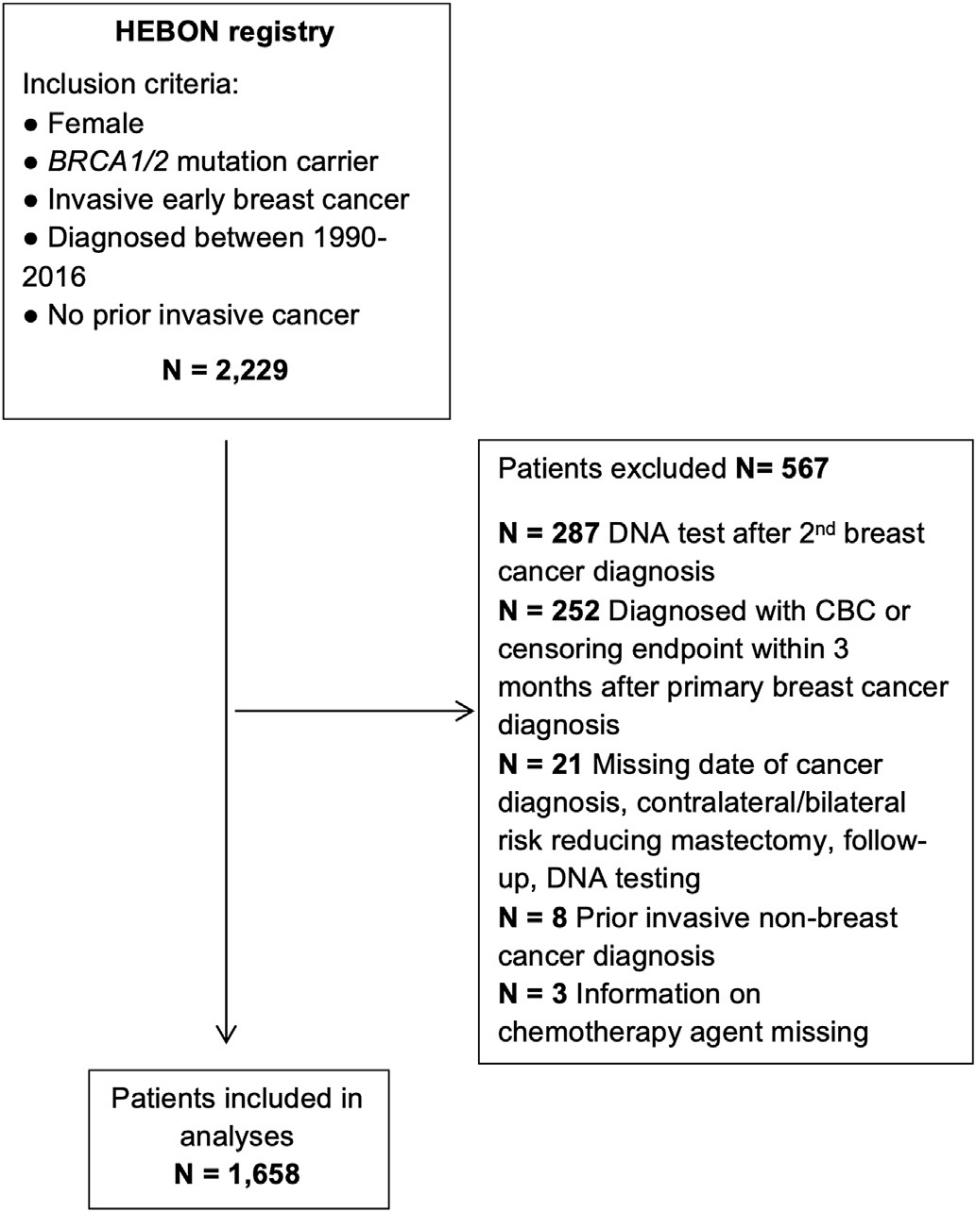
Inclusion of participants.Abbreviations: CBC = contralateral breast cancer; HEBON = Hereditary Breast and Ovarian Cancer Research Netherlands.

We selected women with a proven pathogenic germline *BRCA1* or *BRCA2* mutation, diagnosed with invasive stage I-III PBC between 1990 and July 2017 (Fig. A.1). Information on patient, tumour, treatment and follow-up characteristics was obtained. Patients were excluded if they had a history of invasive cancer prior to their PBC (except non-melanoma skin cancer) or if data were missing regarding PBC diagnosis, chemotherapy (yes vs. no) or follow-up (i.e. dates of cancer diagnosis, DNA test results, risk-reducing surgeries, or death).

## 3 Statistical analysis

The primary endpoint was the development of a metachronous CBC, defined as the development of a new invasive or in situ tumour in the contralateral breast at least 3 months after PBC diagnosis. We assessed the effect of neo-adjuvant or adjuvant chemotherapy overall, and of different chemotherapeutic agents, compared to no chemotherapy, on metachronous CBC risk. The secondary outcome was exclusively invasive CBC.

We performed two separate analyses to determine CBC risk: 1. competing risk analysis was used to determine cumulative incidence for CBC with death and contralateral or bilateral risk-reducing mastectomy as competing risks; 2. the Cox proportional hazards model was used to estimate cause-specific hazard ratios (HRs) and 95% confidence intervals (95% CI) for the association of chemotherapy with CBC risk with death and contralateral or bilateral risk-reducing mastectomy as censoring endpoints. In both the competing risk and the cause-specific analyses, additional censoring endpoints were secondary invasive cancer diagnosis (except non-melanoma skin cancer), ipsilateral secondary invasive/non-invasive breast cancer diagnosis or end of study (12/31/2017).

Age at PBC, radiotherapy, adjuvant endocrine therapy, risk-reducing salpingo-oophorectomy (time-dependent) and TNM-stage were considered as potential confounders based on published literature. Since metachronous CBC was defined as the development of a tumour in the contralateral breast at least 3 months following a PBC diagnosis, follow-up started from 3 months onwards for all patients (i.e., patients with an endpoint within 3 months were excluded). To account for prevalent cases, we applied left truncation; follow-up started 3 months after PBC diagnosis or at DNA test result, whichever came last.

For the overall analysis on chemotherapy vs. no chemotherapy, 10-year HRs were provided (i.e., patients were censored at 10 years). This cut-off was set to take into account the median follow-up. Time-dependency was explored by comparing HR estimates for the first 5 years versus 5–10 years of follow-up.

For the different chemotherapy agents, 5-year HRs were provided in order to account for the shorter median follow-up of the patients who received more recent types of treatment. Chemotherapy was categorized into 3 mutually exclusive groups: 1. CMF: cyclophosphamide, methotrexate and 5-fluorouracil (5-FU); 2. Anthracyclines and/or platinum-based agents; 3. Combinations of anthracyclines and taxanes, with or without platinum-based agents. Chemotherapeutic agents were unknown in 40% of the cases (Supplementary Material A.4-A.5). We imputed unknown agents, as we know from literature that imputation can provide more reliable results than performing a complete case analysis [[15], [16], [17]]. Because agents depended strongly on year of PBC diagnosis, age at PBC diagnosis, PBC hormone receptor status, tumour grade and TNM-stage (according to the Dutch guidelines [18]), we performed mode imputation stratified by these variables as well as hospital of treatment and the distribution of different chemotherapy agents over the years. Patients were categorized as having received CMF if PBC diagnosis was before January 01, 1994; anthracyclines if PBC diagnosis was between 12/31/1997 and January 01, 2007; and anthracyclines in combination with taxanes if PBC diagnosis was from January 01, 2009 onwards. We additionally confirmed whether imputed agents were equal to known agents of comparable patients from the same hospital, i.e. diagnosed with PBC in the same year and with comparable TNM-stage and age at PBC diagnosis. A sensitivity analysis without imputation of chemotherapeutic agents (i.e. complete case analysis) was performed and compared with the main analysis.

For radiotherapy and endocrine therapy, missing values (28 patients in total) were imputed for the Cox model, based on other treatment determining characteristics or, if not possible, using cold deck imputation. For the purpose of comparison with previous studies, we also obtained combined *BRCA1* and *BRCA2* estimates (Supplementary Tables A.1-A.3).

The proportional hazards assumption was evaluated visually and, if proportional hazards violation of a variable was suspected, through adding an interaction term with time. Interaction testing was performed between chemotherapy and *BRCA* carrier status and between chemotherapy as categorized into 3 groups and *BRCA* carrier status to check for formal evidence of differential effect. Statistical analyses were performed using Stata (version 16).

## 4 Results

In total, 1090 *BRCA1* and 568 *BRCA2* mutation carriers were included (Table A.1). Median follow-up was 8.9 years for *BRCA1* and 8.5 years for *BRCA2* mutation carriers.

CBC was observed as the first event in 116 *BRCA1* and 44 *BRCA2* mutation carriers, of which 23 and 18 were non-invasive, respectively. In 757 patients, risk-reducing mastectomy was performed prior to another event. Death was observed in 244 patients as the first event.

### 4.1 Cumulative CBC risk

**Fig. A.2:**
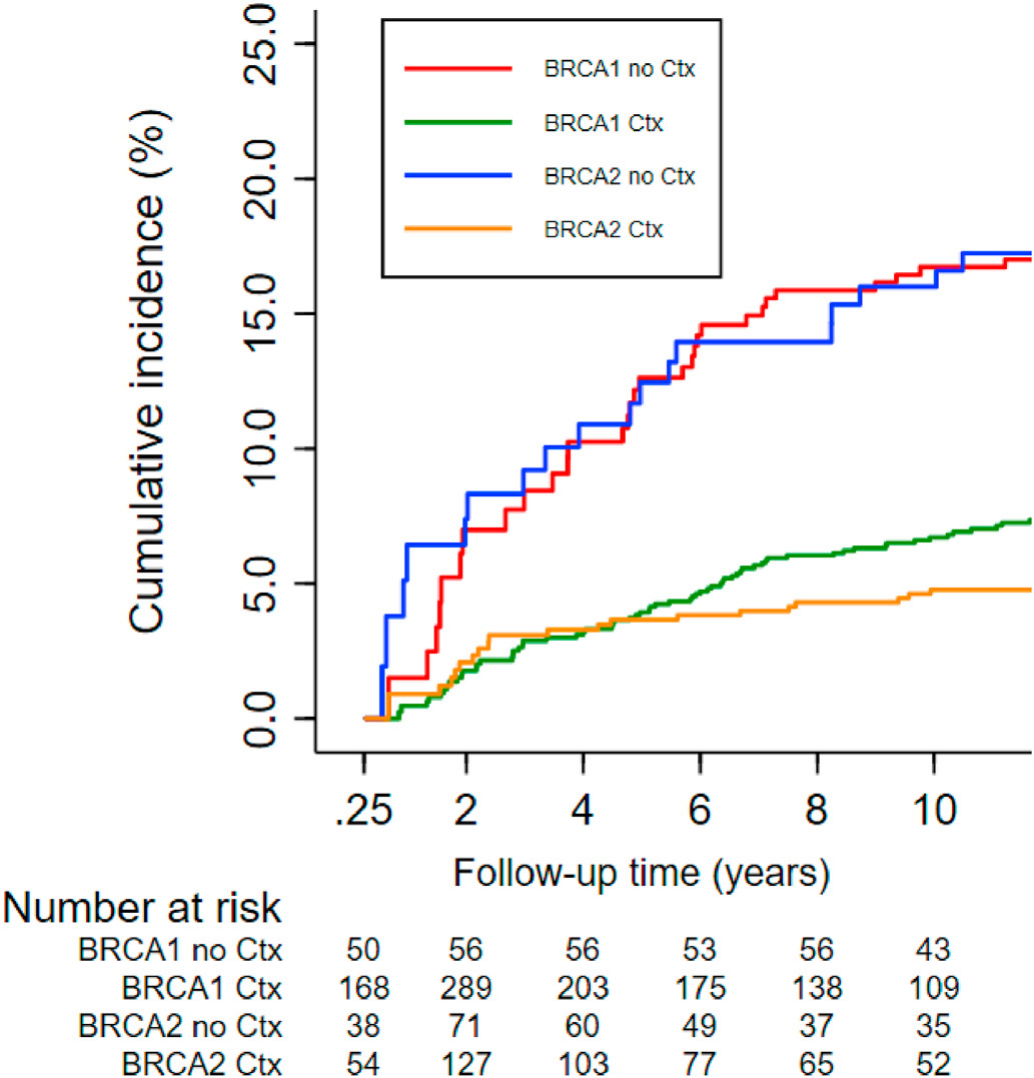
Cumulative incidence of developing CBC in *BRCA1* and *BRCA2* mutation carriers (%); chemotherapy vs. no chemotherapy. Abbreviations: CBC = contralateral breast cancer; Ctx = chemotherapy. Competing risk analysis were applied for this figure.

**Fig. A.3A:**
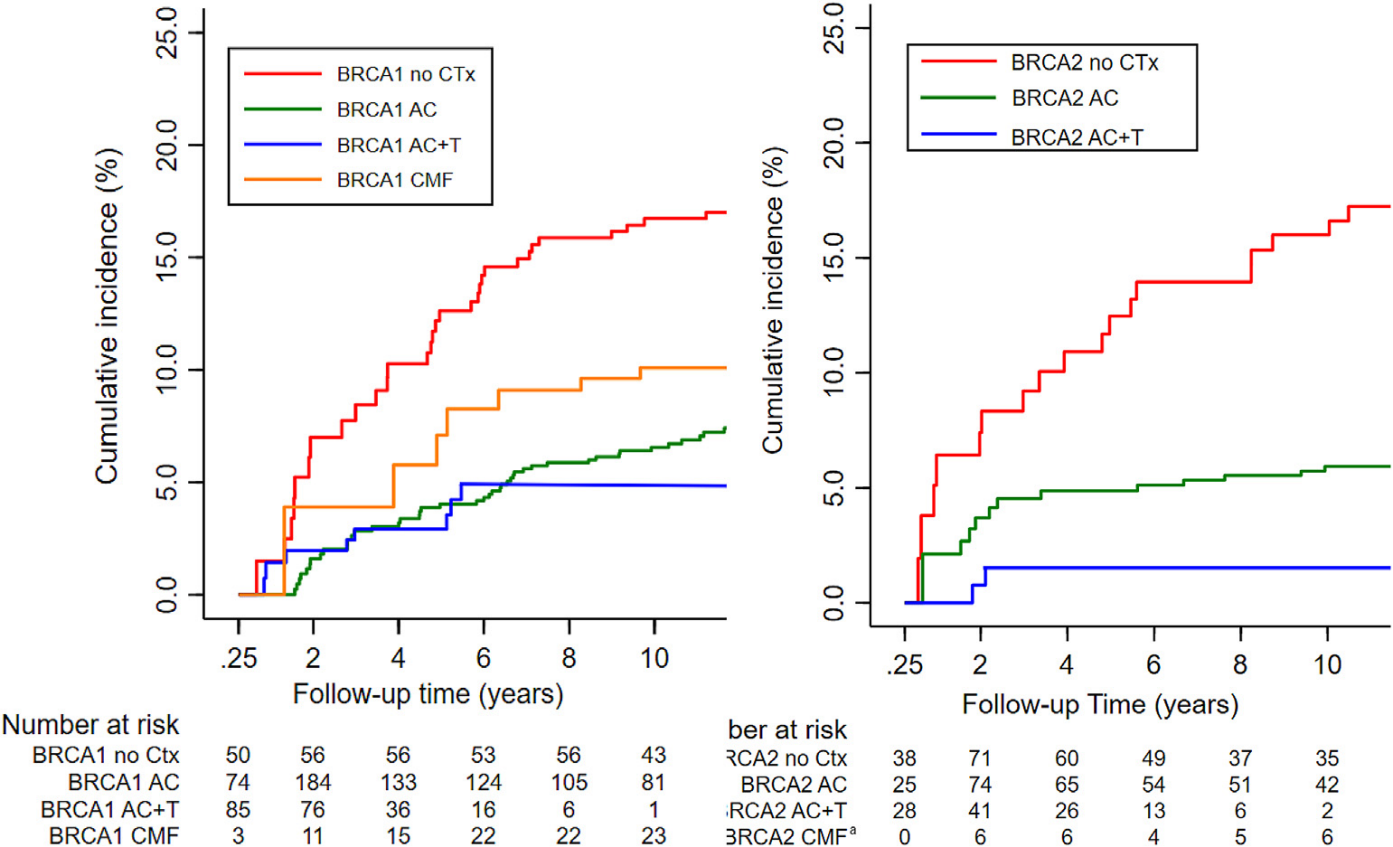
(left) Cumulative incidence of developing CBC in *BRCA1* mutation carriers (%); Anthracyclines vs. Anthracyclines + Taxanes vs. CMF vs. no chemotherapy. Abbreviations: AC = Anthracyclines; AC + T = Anthracyclines + Taxanes; CMF= Cyclophosphamide Methotrexate and 5-FU; CBC = contralateral breast cancer; Ctx = chemotherapy. Competing risk analysis were applied for this figure. Fig. A.3B(right) Cumulative incidence of developing CBC in *BRCA2* mutation carriers (%); Anthracyclines vs. Anthracyclines + Taxanes vs. no chemotherapy. Abbreviations: AC = Anthracyclines; AC + T = Anthracyclines + Taxanes; CBC = contralateral breast cancer; Ctx = chemotherapy. Competing risk analysis were applied for this figure. ^a^CMF was left out because limited events (n = 1).

Ten-year cumulative CBC risk for *BRCA1* mutation carriers was 6.7% [95% CI: 5.1–8.6] after treatment with chemotherapy and 16.7% [95% CI: 10.8–23.7] without chemotherapy. In *BRCA2* mutation carriers, the 10-year cumulative incidence rates were 4.8% [95% CI: 2.7–7.8] and 16.0% [9.3–24.4], respectively (Table A.2and Fig. A.2). All subtypes of chemotherapy were associated with reduced CBC risk in *BRCA1* mutation carriers, although CMF appears less effective than anthracyclines and taxanes (Fig. A.3A). For *BRCA2* mutation carriers similar trends were observed when comparing the different agents (Fig. A.3B).

### 4.2 Chemotherapy vs. no chemotherapy

For *BRCA1* mutation carriers, treatment with neo-adjuvant or adjuvant chemotherapy compared to no chemotherapy was associated with decreased CBC risk (multivariable 10-year HR: 0.46, 95% CI: 0.29–0.74; Table A.3). We mainly observed a risk-reducing effect of chemotherapy in the first five years after PBC (HR: 0.32, 95% CI: 0.17–0.61 for the first five years after PBC diagnosis and HR: 0.69, 95% CI: 0.35–1.37 for five years onwards; p-value = 0.27 for trend; Fig. A.2). For *BRCA2* mutation carriers, a similar trend in 10-year risk reduction was observed (multivariable HR: 0.63, 95% CI: 0.29–1.39; Table A.3; p-value = 0.44 for interaction for differences in associations between *BRCA1* and *BRCA2* patients).

### 4.3 Chemotherapy agents

For B*RCA1* mutation carriers, treatment with anthracyclines was specifically associated with reduced CBC risk (multivariable HR: 0.34, 95% CI: 0.17–0.67; Table A.4). We observed similar effects for combinations of anthracyclines and taxanes (multivariable HR: 0.22, 95% CI: 0.08–0.62; Table A.4 and Fig. A.3A). We had insufficient power (as indicated by the wide confidence interval) to prove or refute a significant difference between the combination of anthracyclines and taxanes versus treatment with anthracyclines alone (multivariable HR: 0.65, 95% CI: 0.24–1.65). For *BRCA2* mutation carriers similar trends for the chemotherapeutic agents were observed (Table A.4).

Risk estimates for invasive CBC are presented in Supplementary Tables B.1-B.3. For both *BRCA1* and *BRCA2* mutation carriers, cumulative incidences and hazard ratios for invasive CBC were comparable with the combined invasive and non-invasive CBC risk estimates.

Complete case analysis revealed similar results as the main analysis (Supplementary Material A.4-A.6).

## 5 Discussion

We observed a reduced risk of metachronous CBC in *BRCA1* mutation carriers who received chemotherapy compared to those who did not. For *BRCA2* mutation carriers, we observed a similar trend (HR: 0.63, 95% CI: 0.29–1.39). In both groups, there was a large difference in cumulative incidence of CBC by chemotherapy. We are the first to study the effects of different chemotherapeutic agents on CBC risk, separately for *BRCA1* and *BRCA2* mutation carriers. The risk-reducing effects were the largest in *BRCA1* mutation carriers who were treated with anthracyclines alone or in combination with taxanes, though these effects only concern the first 5 years after PBC diagnosis.

In earlier studies [1,11,18], CBC risk reduction after chemotherapy was already described, which is in line with our study. However, only in the study by Reding et al. [11], the effects of different agents were examined. Reding et al. observed a decreased CBC risk, though in a combined cohort of *BRCA1* and *BRCA2* mutation carriers who were treated with anthracyclines versus those who received no chemotherapy. We also observed a risk-reducing effect when we combined *BRCA1* and *BRCA2* mutation carriers. However, in our study the effects were especially prominent among *BRCA1* mutation carriers. The limited number of patients and/or events in *BRCA2* mutation carriers though, preclude strong claims on the impact of chemotherapy in *BRCA2* mutation carriers. Also, in *BRCA2* mutation carriers the impact of endocrine therapy most likely played a more important role. Moreover, while both *BRCA1* and *BRCA2* associated tumours have a homologous recombination repair deficiency, there are phenotypical characteristics which could lead to a different chemotherapeutic response [1,12,13]. In our study for example, *BRCA1* mutation carriers were more often aged under 35 years at PBC diagnosis than *BRCA2* mutation carriers (29.8% vs. 16.7% respectively), more often had grade III PBC (83.7% vs. 56.5%), and more often had ER-negative PBC (78.2% vs. 24.7%). These features are all associated with more aggressive tumour growth and worse prognosis [[19], [20], [21], [22]], and therefore chemotherapy is likely more effective in *BRCA1* mutation carriers (and by extension in the prevention of secondary breast tumours, having similar characteristics, at least in our dataset).

Double-strand DNA breaks-inducing chemotherapeutics, e.g. anthracyclines, are more effective in homologous recombination repair deficient (pre-)cancerous cells of *BRCA1/2* mutation carriers, eliminating (pre-)cancerous lesions [23]. Indeed, our limited data suggests that there was a stronger risk-reducing effect of anthracycline-based chemotherapeutics. In earlier studies, tumours in *BRCA1* mutation carriers were found to be less sensitive to taxane-based chemotherapy than tumours of sporadic breast cancer patients [24,25]. Taxanes do not cause double-strand DNA breaks, but act through stabilization of microtubules, resulting in cell-cycle arrest and apoptosis [26]. In a recent study however, no resistance to taxane agents was observed [6]. Taxanes may provide an additional benefit, although in our study numbers were too small to draw a definite conclusion. Further, there have been important developments in treatment over the years, i.e. better dosage of anthracyclines (e.g. dose-dense scheduling), better monitoring and better support during treatment, increasing therapy adherence, which may affect the results.

The cumulative CBC incidences we observed in *BRCA1* and *BRCA2* mutation carriers were comparable with the results from earlier studies [1,2]. We aimed to reduce survival bias by left-truncating the analysis, i.e. person-time prior to DNA testing was not taken into account. This automatically led to exclusion of patients with CBC diagnosis prior to *BRCA1/2* DNA mutation testing (*n* = 287; Fig A.1), preventing an overrepresentation of CBC patients who may have undergone a DNA test because of the CBC diagnosis (i.e. limiting testing bias). This could lead to an overcorrection lowering CBC risk. On the other hand, a proportion of the mutation carriers with breast cancer who did not develop a CBC may not have been identified as a mutation carrier and are therefore not included in the study. The exclusion of these low-risk women will likely have caused an overestimation of the CBC risk in our study population, balancing a potential overcorrection. Further, although the number of exclusions was high, 25% of these patients (n = 73) would still have been excluded for other reasons eventually (mainly synchronous CBC development).

The strengths of our study are the use of a cohort with nationwide coverage and generally long follow-up, as well as being the first study aiming to estimate the effect of different chemotherapeutic agents on CBC risk separately in *BRCA1* and *BRCA2* mutation carriers.

Still, there are some limitations to our study. Ideally a randomized trial would be performed to investigate the effect of systemic treatment on CBC risk. However, it would be unethical to withhold chemotherapy from patients who are presumed to benefit from it. Therefore, we made use of existing data from an observational study. By taking into account selection and survival bias we attempted to approach a prospective study design as much as possible. Finally, around 40% of the data on chemotherapy agents was initially missing, which could have influenced the results. However, after imputation, missing data was limited to 8%, and we observed no relevant differences when comparing the results including versus excluding the imputed agents.

Another potential limitation was the imbalance in the risk-reducing mastectomy rates between patients who were treated with chemotherapy vs. without chemotherapy (higher in the chemotherapy group). This could potentially lead to a bias. We observed that in *BRCA1* mutation carriers the median time from primary breast cancer diagnosis until a DNA test, was much shorter in the chemotherapy group (1.0 year) than in the non-chemotherapy group (3.4 years). Both the earlier DNA testing and the increased risk-reducing mastectomy rates are suggestive of a stronger family history with an even higher CBC incidence rate within these families, indicating that the baseline risk of CBC was higher in this group. Consequently, the actual CBC rate in the chemotherapy group should have been higher than we described, suggesting the protective effect of chemotherapy on CBC risk we observed is an underestimation.

## 6 Clinical implications

The primary goal of chemotherapy is to eliminate micrometastases and reducing the risk of distant and loco-regional recurrences. By extension, this may also eliminate preclinical/precancerous lesions in the contralateral breast and in that case, the effect will likely be transient. Indeed, in this study we now showed also a strong risk-reducing effect of chemotherapy in the first 5 years after PBC diagnosis on the development of new primary cancers in the contralateral breast, most notably in *BRCA1* mutation carriers. Since patients with *BRCA1-*and *BRCA2*-associated PBC have a high baseline risk of developing CBC, the relative benefit of chemotherapy leads to a high absolute reduction in CBC risk. The results of our study can be used to further personalise CBC risk management. In combination with other factors that influence CBC risk [4], we aim to identify patients at high and low risk of CBC [27,28]. Based on the results of this study, the frequency of screening and choices regarding risk-reducing surgeries cannot be tailored to the different risk-profiles yet, though this would be the subsequent goal. Hereto, long-term effects of chemotherapy on CBC risk (i.e. beyond our median follow-up of ten years), should be investigated first, in particular in young *BRCA1/2* mutation carriers with long life expectancy. After all, if after ten years, the annual CBC risk normalises to the level of those without chemotherapy (i.e., 1.5–3.0%), overall lifetime CBC risk would not be lowered enough to change decision-making regarding screening or risk-reducing surgery. Furthermore, in future studies, the long-term effects of more recent developments in drug treatment (e.g. PARP-inhibitors), should also be taken into account.

## 7 Conclusions

Chemotherapy is associated with reduced CBC risk in BRC*A1* mutation carriers at least for the first 5 years. Anthracyclines, either alone or in combination with taxanes, may result in the largest risk reduction. For *BRCA2* mutation carriers, results pointed in the same direction. The risk-reducing effects of chemotherapy can be used to further personalise CBC risk assessment.

## Funding

This study was funded by the Dutch Cancer Society/Alpe d’HuZes (Grant Number: A6C/6253).

## Declaration of competing interest

None.

**Table A.1:** Characteristics of *BRCA1* and *BRCA2* PBC patients: chemotherapy versus no chemotherapy.

	*BRCA1*						*BRCA2*					Total Group	
	No Chemotherapy		Chemotherapy b			p-value	No chemotherapy		Chemotherapy b		p-value		
	N	%	N	%			N	%	N	%		N	%
Total	*276*	*25.3*	*814*	*74.7*			*191*	*33.6*	*377*	*66.4*		*1658*	100

Median FU in years [range]	13.8 [0.3–27.9]		10.0 [0.4–27.7]			<0.001	10.4 [0.5–26.8]		9.7 [0.8–26.3]		0.4043	10.3 [0.3–27.9]	
FU in years after left truncation [range]	10.5 [0.3–26.5]		8.5 [0.4–23.6]			<0.001	8.5 [0.5–24.2]		8.4 [0.8–25.6]		0.3037	8.8 [0.3–26.5]	

Age at PBC													
Median age, years [range]	46.5 [22–85]		39.4 [19–70]			<0.001	52.5 [24–87]		43.3 [20–70]		<0.001	42.2 [19–87]	
						<0.001					<0.001		
<30	15	5.5	84	10.3			4	2.1	13	3.5		116	7.0
30-34	26	9.5	163	20.0			8	4.2	47	12.5		244	14.7
35-39	40	14.6	189	23.2			23	12.0	76	20.2		328	19.8
40-44	41	14.9	153	18.8			17	8.9	82	21.8		293	17.7
45-49	48	17.5	107	13.1			24	12.6	68	18.0		247	14.9
50-54	35	12.7	67	8.2			38	19.9	46	12.2		186	11.2
55-59	23	8.4	29	3.6			21	11.0	27	7.2		100	6.0
60+	47	17.1	22	2.7			56	29.3	18	4.8		143	8.6
Unknown	1		0				0		0			1	

Year of PBC diagnosis						<0.001					<0.001		
1990–1994	87	31.5	61	7.5			30	15.7	21	5.6		199	12.0
1995–1999	90	32.6	122	15.0			36	18.9	44	11.7		292	17.6
2000–2004	37	13.4	223	27.4			49	25.7	115	30.5		424	25.6
2005–2009	42	15.2	284	34.9			50	26.2	143	37.9		519	31.3
2010–2017	20	7.3	124	15.2			26	13.6	54	14.3		224	13.5

Stage^a^						<0.001					<0.001		
IA	153	64.8	223	31.0			114	65.1	66	19.6		556	37.9
IB	4	1.7	20	2.8			5	2.9	9	2.7		38	2.6
IIA	64	27.1	264	36.7			35	20.0	88	26.2		451	30.7
IIB	8	3.4	130	18.1			14	8.0	82	24.4		234	16.0
IIIA	2	0.9	54	7.5			4	2.3	48	14.3		108	7.4
IIIB	3	1.3	9	1.3			0	0	9	2.7		21	1.4
IIIC	2	0.9	20	2.8			3	1.7	34	10.1		59	4.0
Unknown	40		94				16		41			191	

Histological B&R grade						<0.001					<0.001		
Grade I	7	3.5	8	1.1			14	9.0	8	2.5		37	2.7
Grade II	57	28.6	77	10.8			74	47.7	113	34.8		321	23.0
Grade III	135	67.8	630	88.1			67	43.2	204	62.8		1036	74.3
Unknown	77		99				36		52			264	

Oestrogen receptor status					<0.001						0.083		
Positive	57	36.5	133	18.9			115	80.4	240	72.7		545	40.9
Negative	99	63.5	572	81.1			28	19.6	90	27.3		789	59.2
Unknown	120		109				48		47			324	

Progesterone receptor status					0.017						0.603		
Positive	37	25.3	112	16.5			80	59.7	179	56.8		408	32.1
Negative	109	74.7	565	83.5			54	40.3	136	43.2		864	67.9
Unknown	130		137				57		62			386	

HER2 receptor status					0.197						0.204		
Positive	7	9.5	28	5.6			5	5.5	26	10.9		66	7.3
Negative	67	90.5	469	94.4			86	94.5	213	89.1		835	92.7
Unknown	202		317				100		138			757	

Surgery					0.022						<0.001		
None/biopsy	4	1.6	11	1.4			7	3.9	12	3.2		34	2.1
Lumpectomy	135	54.4	359	44.9			102	56.4	121	32.3		717	44.7
Mastectomy	109	44.0	429	53.7			72	39.8	242	64.5		852	53.2
Unknown	28		15				10		2			55	

Radiotherapy					0.165						0.516		
Yes	150	57.5	507	62.4			108	59.3	234	62.4		999	61.3
No	111	42.5	305	37.6			74	40.7	141	37.6		631	38.7
Unknown	15		2				9		2			28	

Endocrine therapy					<0.001						<0.001		
Yes	31	11.9	178	21.9			49	26.9	237	62.9		495	30.3
No	230	88.1	634	78.1			133	73.1	140	37.1		1137	69.7
Unknown	15		2				9		0			26	

Targeted therapy					c						c		
Yes	0	0	27	3.3			0	0	24	6.4		51	3.1
No	261	100	785	96.4			182	100	353	93.6		1581	96.9
Unknown	15		2				9		0			26	

CRRM/BRRM					<0.001						<0.001		
Yes	94	34.1	457	56.1			55	28.8	215	57.0		821	49.5
No	182	65.9	357	43.9			136	71.2	162	43.0		837	50.5

RRSO					<0.001						<0.001		
Yes	173	63.1	634	78.7			122	64.2	306	81.4		1235	75.0
No	101	36.9	172	21.3			68	35.8	70	18.6		411	25.0
Other/Unknown	2		8				1		1			12	

Abbreviations: B&R = Bloom & Richardson; BRRM = bilateral risk-reducing mastectomy; CRRM = contralateral risk-reducing mastectomy; FU = follow-up; PBC = primary breast cancer; RRSO = risk-reducing salpingo oophorectomy.

Differentiation grade: grade I = well differentiated; grade II = moderately differentiated; grade III = poorly differentiated/undifferentiated. Missing values were excluded for the Chi-square/Kruskal-Wallis significance testing of the variables.

a Pathological TNM was used to determine stage, except for patients who received neo-adjuvant chemotherapy, clinical TNM-stage was used. Stages: IA = T1 N0 M0; IB = T0-1 N1mi M0; IIA = T0-1 N1 M0 or T2 N0 M0; IIB = T2 N1 M0 or T3 N0 M0; IIIA = T0-2 N2 M0 or T3 N1-2 M0; IIIB = T4 N0-2 M0; IIIC = Any T N3 M0.

b Neo-adjuvant or adjuvant chemotherapy (93 vs. 748 in *BRCA1* and 57 vs. 320 in *BRCA2*, respectively).

c No significance testing was performed since targeted therapy was always provided in combination with chemotherapy.

**Table A.2:** Five- and ten-year cumulative incidence of metachronous CBC in *BRCA1* and *BRCA2* mutation carriers: chemotherapy vs. no chemotherapy.

	N CBC/N PBC	5-year CBC risk % [95% CI]	10-year CBC risk % [95% CI]
***BRCA1* mutation carriers**			
Total	116/963	**5.2** [3.8–7.0]	**8.2** [6.5–10.1]
Chemotherapy	79/749	**3.9** [2.7–5.6]	**6.7** [5.1–8.6]
No chemotherapy	37/214	**12.6** [7.3–19.4]	**16.7** [10.8–23.7]
***BRCA2* mutation carriers**			
Total	44/506	**6.3** [3.9–9.7]	**8.1** [5.4–11.4]
Chemotherapy	23/344	**3.7** [1.8–6.6]	**4.8** [2.7–7.8]
No chemotherapy	21/162	**12.5** [6.4–20.7]	**16.0** [9.3–24.4]

Abbreviations: CBC = contralateral breast cancer, either invasive or non-invasive; CI = confidence interval; PBC = primary breast cancer.

Competing risk analysis was used to determine cumulative incidence for invasive CBC.

**Table A.3:** Univariable and multivariable Cox regression analyses for 10-year risk of metachronous CBC, stratified by *BRCA1* and *BRCA2* mutation

	PYO	N CBC	Rate Per 1000 PYO	uHR [95% CI]	mHR [95% CI]
***BRCA1* mutation carriers**					
**Chemotherapy**	1939	59	30.4	0.56 [0.36–0.88]	0.46 [0.29–0.74]
No chemotherapy	538	29	53.9	**Ref.**	**Ref.**
**Endocrine therapy**	540	14	25.9	0.68 [0.38–1.20]	0.78 [0.44–1.40]
No endocrine therapy	1937	74	38.2	**Ref.**	**Ref.**
**Radiotherapy**	1716	64	37.3	1.04 [0.65–1.67]	1.10 [0.68–1.77]
No Radiotherapy	760	24	31.6	**Ref.**	**Ref.**
**Age** (continuous)	2477	88	35.5	0.98 [0.96–1.00]	0.97 [0.95–0.99]
***BRCA2* mutation carriers**					
**Chemotherapy**	869	19	21.9	0.70 [0.36–1.37]	0.63 [0.29–1.39]
No chemotherapy	512	16	31.2	**Ref.**	**Ref.**
**Endocrine therapy**	772	13	16.8	0.48 [0.24–0.95]	0.53 [0.25–1.12]
No endocrine therapy	610	22	36.1	**Ref.**	**Ref.**
**Radiotherapy**	925	24	26.0	1.11 [0.54–2.28]	1.17 [0.57–2.42]
No radiotherapy	457	11	24.1	**Ref.**	**Ref.**
**Age** (continuous)	1381	35	25.3	0.97 [0.94–1.00]	0.96 [0.93–0.99]

Abbreviations: PYO = person-years of observation; N CBC = number of contralateral breast cancer events, either invasive or non-invasive; uHR = univariable hazard ratios; mHR = multivariable hazard ratios, with adjustment for all other variables in the model (e.g. chemotherapy was adjusted for endocrine therapy, radiotherapy and age; age was adjusted for chemotherapy, endocrine therapy and radiotherapy).

Adjusting for risk-reducing salpingo oophorectomy (time-dependent) did not lead to a substantial change in the hazard ratio and was therefore not included the multivariable model.

Age concerns age at primary breast cancer diagnosis.

**Table A.4:** Univariable and multivariable Cox regression analyses for 5-year risk of metachronous CBC according to different partly imputed chemotherapy agents, stratified by *BRCA1* and *BRCA2* mutation.

	PYO	N CBC	Rate Per 1000 PYO	uHR [95% CI]	mHR [95% CI]
***BRCA1* mutation carriers**					
**Anthracyclines**	724	20	27.6	0.42 [0.22–0.81]	0.34 [0.17–0.68]
Anthracyclines + Taxanes	319	5	15.7	0.28 [0.10–0.76]	0.22 [0.08–0.62]
CMF	69	3	43.6	0.65 [0.19–2.22]	0.57 [0.16–1.95]
No chemotherapy	274	17	62.1	**Ref.**	**Ref.**
**Endocrine therapy**	332	10	30.1	0.93 [0.46–1.87]	1.12 [0.54–2.30]
No endocrine therapy	1140	37	32.4	**Ref.**	**Ref.**
**Age** (continuous)	1472	47	31.9	0.99 [0.96–1.02]	0.98 [0.95–1.00]
***BRCA2* mutation carriers**					
**Anthracyclines**	294	7	23.8	0.68 [0.26–1.76]	0.64 [0.22–1.86]
Anthracyclines + Taxanes	177	2	11.3	0.30 [0.07–1.36]	0.30 [0.06–1.51]
CMF	21	1	47.1	1.32 [0.17–10.30]	0.80 [0.10–6.56]
No chemotherapy	304	11	36.2	**Ref.**	**Ref.**
**Endocrine therapy**	472	8	17.0	0.41 [0.17–0.96]	0.49 [0.19–1.26]
No endocrine therapy	353	15	42.5	**Ref.**	**Ref.**
**Age** (continuous)	825	23	27.9	0.97 [0.94–1.01]	0.96 [0.92–1.00]

Abbreviations: CMF = Cyclophosphamide Methotrexate and 5-FU; PYO = Person-years of observation; N CBC = number of contralateral breast cancer events, either invasive or non-invasive; uHR = univariable hazard ratios; mHR = multivariable hazard ratios, with adjustment for all other variables included in the model (e.g. chemotherapeutic agents was adjusted for endocrine therapy and age; age was adjusted for chemotherapeutic agents and endocrine therapy).

Adjusting for risk-reducing salpingo oophorectomy (time-dependent) did not lead to a substantial change in the hazard ratio and was therefore not included the multivariable model.

For the missing chemotherapeutic agents, patients were categorized as CMF if the primary breast cancer diagnosis was <January 01, 1994, Anthracyclines if the primary breast cancer diagnosis was between 12/31/1997 and January 01, 2007, and Anthracyclines + Taxanes if the primary breast cancer diagnosis was >12/31/2008.

For Fig. A.2, A.3A and A.3B below colour should be used in print.
