# Supplementary material for: Effects of chemotherapy on contralateral breast cancer risk in BRCA1 and BRCA2 mutation carriers: A nationwide cohort study
Source: Breast. 2021 Dec 14;61:98–107. doi: 10.1016/j.breast.2021.12.007 (PMC8693290; doi:10.1016/j.breast.2021.12.007)
Supplement: Multimedia component 1 [file mmc1.docx]

| **Supplementary Table A.1. Univariable and multivariable Cox regression analyses for 10-year risk of metachronous CBC in *BRCA1* and *BRCA2* mutation carriers combined** | | | | | | | |
| --- | --- | --- | --- | --- | --- | --- | --- |
|  | **PYO** | **N CBC** | **Rate**  **Per 1000 PYO** | **uHR [95% CI]** | **mHR [95% CI]** | |  |
| **Total group** | 3,858 | 123 | 31.9 | - | - | |  |
|  |  |  |  |  |  | |  |
| ***BRCA1 mutation*** | 2,477 | 88 | 35.5 | 1.43 [0.96-2.11] | 1.20 [0.78-1.85] | |  |
| *BRCA2* mutation | 1,381 | 35 | 25.3 | **Ref.** | **Ref.** | |  |
|  |  |  |  |  |  | |  |
| **Chemotherapy** | 2,808 | 78 | 27.8 | 0.65 [0.45-0.93] | 0.49 [0.33-0.74] | |  |
| No chemotherapy | 1,050 | 45 | 42.9 | **Ref.** | **Ref.** | |  |
|  |  |  |  |  |  | |  |
| **Endocrine therapy** | 1,312 | 27 | 20.6 | 0.54 [0.35-0.83] | 0.70 [0.44-1.11] | |  |
| No endocrine therapy | 2,546 | 96 | 37.7 | **Ref.** | **Ref.** | |  |
|  |  |  |  |  |  | |  |
| **Radiotherapy** | 2,641 | 88 | 33.3 | 1.08 [0.73-1.60] | 1.12 [0.75-1.66] | |  |
| No Radiotherapy | 1,217 | 35 | 28.8 | **Ref.** | **Ref.** | |  |
|  |  |  |  |  |  | |  |
| **Age** (continuous) | 3,858 | 123 | 31.9 | 0.97 [0.96-0.99] | 0.97 [0.95-0.98] | |  |
|  |  |  |  |  |  | |  |
| Abbreviations: PYO= person-years of observation; N *CBC* = number of contralateral breast cancer events; uHR= univariable hazard ratios; mHR= multivariable hazard ratios, with adjustment for all other variables included in the model (e.g. chemotherapy was adjusted for *BRCA* status, endocrine therapy, radiotherapy and age; age was adjusted for *BRCA* status, chemotherapy, endocrine therapy and radiotherapy).  Adjusting for risk-reducing salpingo oophorectomy (time-dependent) did not lead to a substantial change in the hazard ratio and was therefore not included the multivariable model.  Age concerns age at primary breast cancer diagnosis.  For the missing chemotherapeutic agents, patients were categorized as CMF if the primary breast cancer diagnosis was <12/31/1994, Anthracyclines if the primary breast cancer diagnosis was between 12/31/1997 and 12/31/2006, and Anthracyclines + Taxanes if the primary breast cancer diagnosis was >12/31/2008. | | | | | |  |  |

| **Supplementary Table A.2. Univariable and multivariable Cox regression analyses for 5-year risk of metachronous CBC according to different chemotherapy agents in *BRCA1* and *BRCA2* mutation carriers combined** | | | | | |
| --- | --- | --- | --- | --- | --- |
|  | **PYO** | **N CBC** | **Rate**  **Per 1000 PYO** | **uHR [95% CI]** | **mHR [95% CI]** |
| **Total group** | 2,297 | 70 | 30.5 | - | - |
|  |  |  |  |  |  |
| ***BRCA1* mutation** | 1,472 | 47 | 31.9 | 1.17 [0.71-1.93] | 1.13 [0.66-1.96] |
| *BRCA2* mutation | 825 | 23 | 27.9 | **Ref.** | **Ref.** |
|  |  |  |  |  |  |
| **No chemotherapy** | 577 | 28 | 48.5 | **Ref.** | **Ref.** |
| Anthracyclines | 1,018 | 27 | 26.5 | 0.53 [0.31-0.90] | 0.40 [0.22-0.71] |
| Anthracyclines + Taxanes | 496 | 7 | 14.1 | 0.30 [0.13-0.70] | 0.23 [0.10-0.55] |
| CMF | 90 | 4 | 44.5 | 0.88 [0.31-2.50] | 0.65 [0.22-1.89] |
|  |  |  |  |  |  |
| **Endocrine therapy** | 803 | 18 | 22.4 | 0.64 [0.37-1.09] | 0.85 [0.48-1.52] |
| No endocrine therapy | 1,493 | 52 | 34.8 | **Ref.** | **Ref.** |
|  |  |  |  |  |  |
| **Age** (continuous) | 2,297 | 70 | 30.5 | 0.98 [0.96-1.01] | 0.97 [0.95-0.99] |
|  |  |  |  |  |  |
| Abbreviations: CMF= Cyclophosphamide Methotrexate and 5-FU; PYO= person-years of observation; N *CBC* = number of contralateral breast cancer events; uHR= univariable hazard ratios; mHR= multivariable hazard ratios, with adjustment for all other variables included in the model (e.g. chemotherapeutic agents was adjusted for *BRCA* status, endocrine therapy and age; age was adjusted for *BRCA* status, chemotherapeutic agents and endocrine therapy).  Adjusting for risk-reducing salpingo oophorectomy (time-dependent) did not lead to a substantial change in the hazard ratio and was therefore not included the multivariable model.  Age concerns age at primary breast cancer diagnosis. | | | | | |

| **Supplementary Table A.3. Univariable and multivariable Cox regression analyses for 5-year risk of metachronous CBC in *BRCA1* and *BRCA*2 mutation carriers combined, according to known chemotherapy agents** | | | | | |
| --- | --- | --- | --- | --- | --- |
|  | **PYO** | **N CBC** | **Rate**  **Per 1000 PYO** | **uHR [95% CI]** | **mHR [95% CI]** |
| **Total group** | 2,297 | 70 | 30.5 | - | - |
|  |  |  |  |  |  |
| ***BRCA1* mutation** | 1,472 | 47 | 31.9 | 1.17 [0.71-1.93] | 1.09 [0.63-1.89] |
| *BRCA2* mutation | 825 | 23 | 27.9 | **Ref.** | **Ref.** |
|  |  |  |  |  |  |
| **No chemotherapy** | 577 | 28 | 48.5 | **Ref.** | **Ref.** |
| Anthracyclines | 644 | 23 | 35.7 | 0.72 [0.41-1.25] | 0.55 [0.30-1.00] |
| Anthracyclines + Taxanes | 338 | 5 | 14.8 | 0.32 [0.12-0.82] | 0.24 [0.09-0.66] |
| CMF | 72 | 4 | 55.4 | 1.10 [0.38-3.13] | 0.81 [0.28-2.34] |
|  |  |  |  |  |  |
| **Endocrine therapy** | 803 | 18 | 22.4 | 0.64 [0.37-1.09] | 0.84 [0.47-1.50] |
| No endocrine therapy | 1,493 | 52 | 34.8 | **Ref.** | **Ref.** |
|  |  |  |  |  |  |
| **Age** (continuous) | 2,297 | 70 | 30.5 | 0.98 [0.96-1.01] | 0.97 [0.95-0.99] |
|  |  |  |  |  |  |
| Abbreviations: CMF= Cyclophosphamide Methotrexate and 5-FU; PYO= person-years of observation; N *CBC* = number of contralateral breast cancer events; uHR= univariable hazard ratios; mHR= multivariable hazard ratios, with adjustment for all other variables included in the model (e.g. chemotherapeutic agents was adjusted for *BRCA* status, endocrine therapy and age; age was adjusted for *BRCA* status, chemotherapeutic agents and endocrine therapy).  Adjusting for risk-reducing salpingo oophorectomy (time-dependent) did not lead to a substantial change in the hazard ratio and was therefore not included the multivariable model.  Age concerns age at primary breast cancer diagnosis. | | | | | |

| **Supplementary Table A.4. Number of initially missing chemotherapy agents (i.e. prior to imputation) in relation to the total number of patients who received chemotherapy in *BRCA1* and *BRCA2* mutation carriers, per period of primary breast cancer diagnosis used for imputing unknown types** |
| --- |

| ***Period of PBC diagnosis*** | ***BRCA1*** | | | ***BRCA2*** | | |
| --- | --- | --- | --- | --- | --- | --- |
|  | ***n/N*** | ***%*** | ***N missings after imputing (%)*** | ***n/N*** | ***%*** | ***N missings after imputing*** |
| 1990-1993 | 48/48 | 100 | 0 | 17/17 | 100 | 0 |
| 1994-1997 | 28/63 | 44.4 | 23 (36.5) | 13/23 | 56.5 | 13 (56.5) |
| 1998-2006 | 145/400 | 36.3 | 0 | 66/193 | 34.2 | 0 |
| 2007-2008 | 43/119 | 36.1 | 40 (33.6) | 24/55 | 43.6 | 18 (32.7) |
| 2009-2017 | 63/184 | 34.2 | 0 | 28/89 | 31.5 | 0 |
| PBC= primary breast cancer; n/N=number of missings/number of patients who received chemotherapy. | | | | | | |

**Supplementary Figure A.5: Number of initially missing chemotherapy agents (prior to imputation) per year of primary breast cancer (PBC) diagnosis* in *BRCA1* and *BRCA2* mutation carriers**


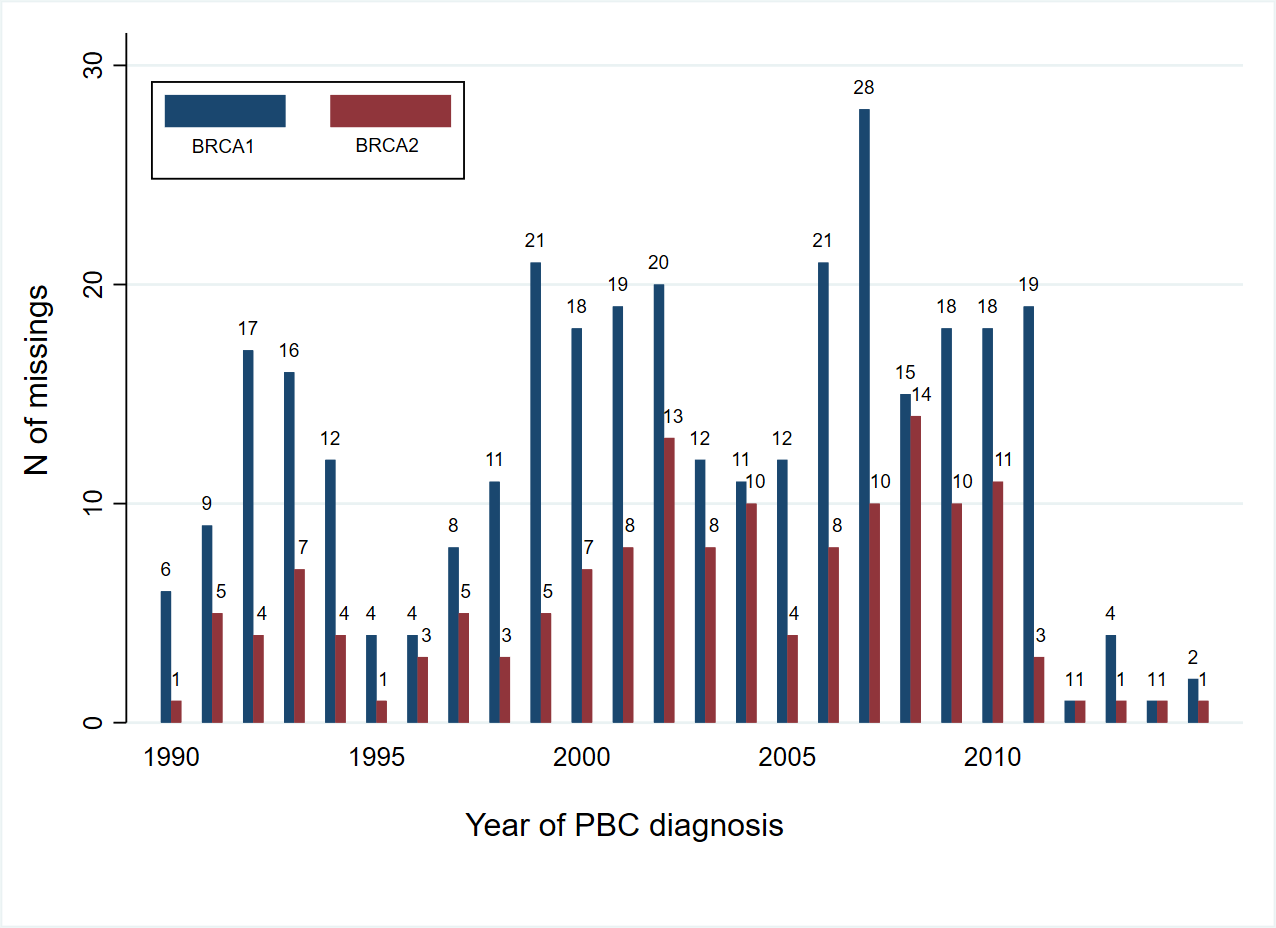


* For 2015 no missings were reported

| **Supplementary Table A.6. Univariable and multivariable Cox regression analyses for 5-year risk of metachronous CBC for different known chemotherapy agents, stratified by *BRCA1* and *BRCA2* mutation carriers** | | | | | |
| --- | --- | --- | --- | --- | --- |
|  | **PYO** | **N CBC** | **Rate per 1000 PYO** | **uHR [95% CI]** | **mHR [95% CI]** |
| ***BRCA1 mutation carriers*** | | | | | |
| **No chemotherapy** | 274 | 17 | 62.1 | **Ref.** | **Ref.** |
| Anthracyclines | 480 | 18 | 37.5 | 0.58 [0.30-1.13] | 0.47 [0.23-0.95] |
| Anthracyclines + Taxanes | 212 | 3 | 14.2 | 0.25 [0.07-0.85] | 0.20 [0.06-0.69] |
| CMF | 52 | 3 | 57.4 | 0.87 [0.25-2.96] | 0.74 [0.21-2.54] |
|  |  |  |  |  |  |
| **Endocrine therapy** | 332 | 10 | 30.1 | 0.93 [0.46-1.87] | 1.11 [0.54-2.28] |
| No endocrine therapy | 1,140 | 37 | 32.4 | **Ref.** | **Ref.** |
|  |  |  |  |  |  |
| **Age** (continuous) | 1,472 | 47 | 31.9 | 0.99 [0.96-1.02] | 0.97 [0.95-1.00] |
|  |  |  |  |  |  |
| ***BRCA2* mutation carriers** | | | | | |
|  |  |  |  |  |  |
| **No chemotherapy** | 304 | 11 | 36.2 | **Ref.** | **Ref.** |
| Anthracyclines | 163 | 5 | 30.6 | 0.88 [0.31-2.53] | 0.92 [0.29-2.90] |
| Anthracyclines + Taxanes | 126 | 2 | 15.8 | 0.42 [0.09-1.88] | 0.44 [0.09-2.26] |
| CMF | 20 | 1 | 50.1 | 1.39 [0.18-10.78] | 0.79 [0.10-6.45] |
|  |  |  |  |  |  |
| **Endocrine therapy** | 472 | 8 | 17.0 | 0.41 [0.17-0.96] | 0.45 [0.17-1.17] |
| No endocrine therapy | 353 | 15 | 42.5 | **Ref.** | **Ref.** |
|  |  |  |  |  |  |
| **Age** (continuous) | 825 | 23 | 27.9 | 0.97 [0.94-1.01] | 0.96 [0.92-1.00] |
|  |  |  |  |  |  |
| Abbreviations: CMF= Cyclophosphamide Methotrexate and 5-FU; PYO= person-years of observation; N *CBC* = number of contralateral breast cancer events; uHR= univariable hazard ratios; mHR= multivariable hazard ratios, adjusted for all other variables included in the model (e.g. chemotherapeutic agents was adjusted for endocrine therapy and age; age was adjusted for chemotherapeutic agents and endocrine therapy). Adjusting for risk-reducing salpingo oophorectomy (time-dependent) did not lead to a substantial change in the hazard ratio and was therefore not included the multivariable model.  Age concerns age at primary breast cancer diagnosis. | | | | | |
