# Supplementary material for: Effects of chemotherapy on contralateral breast cancer risk in BRCA1 and BRCA2 mutation carriers: A nationwide cohort study
Source: Breast. 2021 Dec 14;61:98–107. doi: 10.1016/j.breast.2021.12.007 (PMC8693290; doi:10.1016/j.breast.2021.12.007)
Supplement: Multimedia component 2 [file mmc2.docx]

| **Supplementary Table B.1. Five- and ten-year cumulative incidence of metachronous invasive CBC in *BRCA1* and *BRCA2 mutation carriers*: chemotherapy vs. no chemotherapy** | | | |
| --- | --- | --- | --- |
|  | **N CBC / N PBC** | **5-year CBC risk % [95% CI]** | **10-year CBC risk % [95% CI]** |
| ***BRCA1*** | | | |
|  | | | |
| *Total* | 93/963 | **4.0** [2.8-5.4] | **6.4** [5.0-8.1] |
| *Chemotherapy* | 63/749 | **2.9** [1.9-4.3] | **5.2** [3.8-6.9] |
| *No chemotherapy* | 30/214 | **10.3** [5.5-16.8] | **13.9** [8.5-20.6] |
|  | | | |
| ***BRCA2*** | | | |
|  |  |  |  |
| *Total* | 26/506 | **2.9** [1.4-5.3] | **4.1** [2.4-6.5] |
| *Chemotherapy* | 14/344 | **1.1** [0.4-2.5] | **1.9** [0.9-3.5] |
| *No chemotherapy* | 12/162 | **7.9** [3.3-15.1] | **10.2** [5.0-17.6] |
|  | | | |
| Abbreviations: CBC= contralateral breast cancer, only invasive; CI= confidence interval; PBC= primary breast cancer. Competing risk analysis was used to determine cumulative incidence for invasive CBC. | | | |

| **Supplementary Table B.2. Univariable and multivariable Cox regression analyses for 10-year risk of metachronous invasive CBC, stratified by *BRCA1* and *BRCA2*** **mutation** | | | | | | |
| --- | --- | --- | --- | --- | --- | --- |
|  | **PYO** | **N CBC** | **Rate**  **Per 1000 PYO** | **uHR [95% CI]** | **mHR [95% CI]** |  |
| ***BRCA1* mutation carriers** | | | | | |  |
|  |  |  |  |  |  |  |
| **Chemotherapy** | 1,939 | 48 | 24.8 | 0.59 [0.36-0.97] | 0.50 [0.29-0.84] |  |
| No chemotherapy | 538 | 23 | 42.8 | **Ref.** | **Ref.** |  |
|  |  |  |  |  |  |  |
| **Endocrine therapy** | 540 | 11 | 20.4 | 0.66 [0.35-1.26] | 0.73 [0.38-1.41] |  |
| No endocrine therapy | 1,937 | 60 | 31.0 | **Ref.** | **Ref.** |  |
|  |  |  |  |  |  |  |
| **Radiotherapy** | 1,716 | 53 | 30.9 | 1.12 [0.66-1.93] | 1.20 [0.70-2.07] |  |
| No Radiotherapy | 760 | 18 | 23.7 | **Ref.** | **Ref.** |  |
|  |  |  |  |  |  |  |
| **Age** (continuous) | 2,477 | 71 | 28.7 | 0.98 [0.96-1.01] | 0.98 [0.95-1.00] |  |
|  |  |  |  |  |  |  |
| ***BRCA2* mutation carriers** | | | | | |  |
|  |  |  |  |  |  |  |
| **Chemotherapy** | 869 | 10 | 11.5 | 0.60 [0.25-1.44] | 0.61 [0.22-1.70] |  |
| No chemotherapy | 512 | 10 | 19.5 | **Ref.** | **Ref.** |  |
|  |  |  |  |  |  |  |
| **Endocrine therapy** | 772 | 6 | 7.8 | 0.35 [0.13-0.91] | 0.40 [0.14-1.15] |  |
| No endocrine therapy | 610 | 14 | 23.0 | **Ref.** | **Ref.** |  |
|  |  |  |  |  |  |  |
| **Radiotherapy** | 925 | 13 | 14.1 | 0.90 [0.36-2.27] | 0.96 [0.38-2.43] |  |
| No radiotherapy | 457 | 7 | 15.3 | **Ref.** | **Ref.** |  |
|  |  |  |  |  |  |  |
| **Age** (continuous) | 1,381 | 20 | 14.5 | 0.97 [0.93-1.01] | 0.96 [0.92-1.00] |  |
|  |  |  |  |  |  |  |
| Abbreviations: PYO= person-years of observation; N *CBC* = number of invasive contralateral breast cancer events; uHR= univariable hazard ratios; mHR=multivariable hazard ratios, with adjustment for all other variables in the model (e.g. chemotherapy was adjusted for endocrine therapy, radiotherapy and age; age was adjusted for chemotherapy, endocrine therapy and radiotherapy). Adjusting for risk-reducing salpingo oophorectomy (time-dependent) did not lead to a substantial change in the hazard ratio and was therefore not included the multivariable model.  Age concerns age at primary breast cancer diagnosis. | | | | | |  |

| **Supplementary Table B.3. Univariable and multivariable Cox regression analyses for 5-year risk of metachronous invasive CBC according to different partly imputed chemotherapy agents, stratified for *BRCA1* and *BRCA2* mutation** | | | | | |
| --- | --- | --- | --- | --- | --- |
|  | **PYO** | **N CBC** | **Rate**  **Per 1000 PYO** | **uHR [95% CI]** | **mHR [95% CI]** |
| ***BRCA1* mutation carriers** | | | | | |
|  |  |  |  |  |  |
| **Anthracyclines** | 724 | 19 | 26.2 | 0.52 [0.26-1.08] | 0.45 [0.21-0.96] |
| Anthracyclines + Taxanes | 319 | 2 | 6.3 | 0.15 [0.03-0.69] | 0.13 [0.03-0.59] |
| CMF | 69 | 2 | 29.1 | 0.57 [0.13-2.51] | 0.51 [0.11-2.27] |
| No chemotherapy | 274 | 13 | 47.5 | **Ref.** | **Ref.** |
|  |  |  |  |  |  |
| **Endocrine therapy** | 332 | 8 | 24.1 | 0.95 [0.44-2.09] | 1.08 [0.48-2.41] |
| No endocrine therapy | 1,140 | 29 | 25.4 | **Ref.** | **Ref.** |
|  |  |  |  |  |  |
| **Age** (continuous) | 1,472 | 37 | 25.1 | 0.99 [0.97-1.03] | 0.98 [0.95-1.01] |
|  |  |  |  |  |  |
| ***BRCA2* mutation carriers** | | | | | |
|  |  |  |  |  |  |
| **Anthracyclines** | 294 | 1 | 3.4 | 0.15 [0.02-1.21] | 0.25 [0.03-2.16] |
| Anthracyclines + Taxanes | 177 | 2 | 11.3 | 0.53 [0.11-2.56] | 1.10 [0.20-6.06] |
| CMF | 21.2 | 0 | 0 | -^a^ | -^a^ |
| No chemotherapy | 304 | 7 | 23.0 | **Ref.** | **Ref.** |
|  |  |  |  |  |  |
| **Endocrine therapy** | 472 | 1 | 2.1 | 0.07 [0.01-0.54] | 0.08 [0.01-0.65] |
| No endocrine therapy | 353 | 11 | 31.2 | **Ref.** | **Ref.** |
|  |  |  |  |  |  |
| **Age** (continuous) | 825 | 12 | 14.6 | 0.97 [0.92-1.03] | 0.97 [0.92-1.03] |
|  |  |  |  |  |  |
| Abbreviations: CMF= Cyclophosphamide Methotrexate and 5-FU; PYO= person-years of observation; N *CBC* = number of contralateral breast cancer events; uHR= univariable hazard ratios; mHR= multivariable hazard ratios, with adjustment for all other variables in the model (e.g. chemotherapeutic agents was adjusted for endocrine therapy and age; age was adjusted for chemotherapeutic agents and endocrine therapy).  Adjusting for risk-reducing salpingo oophorectomy (time-dependent) did not lead to a substantial change in the hazard ratio and was therefore not included the multivariable model.  ^a^No estimates available. Age concerns age at primary breast cancer diagnosis.  For the missing chemotherapeutic agents, patients were categorized as CMF if the primary breast cancer diagnosis was <01/01/1994, Anthracyclines if the primary breast cancer diagnosis was between 12/31/1997 and 01/01/2007, and Anthracyclines + Taxanes if the primary breast cancer diagnosis was >12/31/2008. | | | | | |
